# Clinical trials involving positron emission tomography and prostate cancer: an analysis of the ClinicalTrials.gov database

**DOI:** 10.1186/s13014-018-1057-3

**Published:** 2018-06-18

**Authors:** Nikola Cihoric, Eugenia Vlaskou Badra, Alexandros Tsikkinis, Vikas Prasad, Stephanie Kroeze, Ivan Igrutinovic, Branislav Jeremic, Marcus Beck, Sebastian Zschaeck, Peter Wust, Pirus Ghadjar

**Affiliations:** 1 Department of Radiation Oncology, Inselspital, Bern University Hospital, University of Bern, Freiburgstrasse 18, 3010 Bern, Switzerland; 2 Department of Nuclear Medicine, University Hospital of Ulm, Ulm, Germany; 3 University of Kragujevac Faculty of Science, Kragujevac, Serbia; 4 BioIRC Center for Biomedical Research, Kragujevac, Serbia; 5 Department of Radiation Oncology, University Hospital Zurich, Zurich, Switzerland; 6 Department of Radiation Oncology, Charité Universitätsmedizin Berlin, Berlin, Germany

**Keywords:** Cancer of prostate, Cancer of the prostate, Neoplasms, prostate, Neoplasms, prostatic, Prostate cancer, Prostate neoplasms, Prostatic cancer, Positron emission tomography, Positron emission, Pet

## Abstract

**Background:**

The goal of this study is to evaluate the status and future perspectives of clinical trials on positron emission tomography in prostate cancer for diagnostic or therapeutic as well as for surveillance purposes.

**Methods:**

The www.ClinicalTrials.gov database was searched on the 20th of January 2017 for all trials containing terms describing “prostate cancer” (prostate, prostatic, malignant, malignancy, cancer, tumor) and “positron emission tomography”. In total 167 trials were identified. Trials that included diseases other than PCa were excluded (*n* = 27; 16%). Furthermore, we excluded trials (*n* = 4, 2%) withdrawn prior to first patient enrollment. The remaining trials (*n* = 137, 82%) were selected for further manual classification analysis.

**Results:**

One hundred thirty-seven trials were detected and analyzed. Majority of trials were in “active” recruitment status (*n* = 46, 34%) followed by trials that had been “completed” - (*n* = 34, 25%) and trials with “closed recruitment but active follow-up” (*n* = 23, 17%). Phase 1 and 2 comprised 46% of the complete trial portfolio. Locally confined disease was of major interest (n = 46, 34%), followed by metastatic disease – not otherwise specified (*n* = 43, 13%). Evaluation of PET was the primary goal of the trial in 114 (83%) cases. Most of the trials evaluated only one agent (*n* = 122, 89%). Choline and PSMA represented two major groups (total 50%) and they were equally distributed across trial portfolio with 25% (*n* = 34) each. PSMA trials showed the highest average annual growth rate of 56%. The trials were conducted in 17 countries.

**Conclusion:**

The scientific community is showing a strong and ever-growing interest in the field and we expect that in the coming years, more phase III trials will be initiated ultimately delivering the required Level 1 evidence.

**Electronic supplementary material:**

The online version of this article (10.1186/s13014-018-1057-3) contains supplementary material, which is available to authorized users.

## Background

Prostate cancer (PCa) represents the most common cancer in men and the third most common cause of cancer death [1]. Most newly diagnosed patients have localized PCa which can be effectively treated using a number of different treatment modalities [1]. Nevertheless, in the high-risk disease setting and in the presence of metastasis [1] despite the usage of a combination of treatment modalities a significant number of PCa patients progress, ultimately leading to death.

For local treatments such as radiation therapy and surgery, exact knowledge of the localization and extend of the disease is a prerequisite for optimal treatment. Current practice requires the usage of multiple imaging modalities for staging but also for optimal treatment selection and planning. As such, multiparametric magnetic resonance imaging (mpMRI) has been shown to be very helpful in assessing the prostate and regional lymph nodes [2]. In addition, positron emission tomography (PET) has been introduced as a functional imaging modality and is usually combined with computed tomography (PET/CT) or magnetic resonance imaging (PET/MRI). Although early experience using the traditional agent, fluorodesoxyglucose (FDG), which has already been successfully used in other tumor entities, was disappointing [3], during the last decade several other agents (e.g. FDG) using different pathways were developed and introduced to our daily clinical practice. Most recently the prostate-specific membrane antigen (PSMA) was recognized as a highly promising target both for diagnostic and therapeutic purposes. Although, sufficient evidence for utilization of PSMA or Choline PET in certain clinical situation is available, because of the associated costs, reimbursement has been an issue in several countries limiting a more extensive usage.

Our daily work and decision-making should ideally be based on results gained through well designed, properly conducted clinical trials, either interventional or observational. Ideally, new knowledge should be reported and disseminated to the scientific community through scientific publications. Unfortunately, this is not always the case and a great number of data remains unpublished with a substantial time being required from completion of a given trial to final publication. For these reasons, numerous governments, international regulatory bodies as well as the international committee of medical journal editors demand registration of the interventional trials in one of the clinical trial registries recognized by the World Health Organization (WHO). Clinical trial registries are valuable tools for information sharing and meta-research [4, 5]. They provide insights into the current research interests, while also pointing to the areas where optimization in the research portfolio is urgently needed [6–8].

As a relatively new entity in the PCa armamentarium, interventional clinical trials on PET are of special interest. A description of conducted and currently ongoing clinical trials will provide clinicians and researchers with the possibility to gain insights into the current state of art of scientific activities.

Therefore, the goal of this study was to evaluate the current status and future perspectives of clinical trials on PET in PCa for diagnostic or therapeutic as well as for surveillance purposes.

## Methods

The www.ClinicalTrials.gov database was searched on the 20th of January 2017 for all trials containing terms describing “prostate cancer” (prostate, prostatic, malignant, malignancy, cancer, tumor) and “positron emission tomography” (positron, PET – case insensitive search). In total 167 trials were retrieved. All available results were downloaded in the form of xml files. Afterwards, a database was designed and all of the data within the xml files were imported to facilitate further data cleaning, classification and management.

Trials that included diseases other than PCa were excluded (*n* = 27; 16%). Furthermore, we excluded trials (*n* = 4, 2%) withdrawn prior to first patient enrolment. The remaining trials (*n* = 137, 82%) were selected for further manual classification analysis.

Firstly, we classified trials according to the primary role of PET in the trial into 2 main categories: 1) Evaluation of PET was the primary goal of a trial, and 2) Utilization and evaluation of PET was optional or a secondary goal of the trial. All reported PET agents were classified based on the mechanisms of action and positron emitter.

Furthermore, we classified the trials according to disease characteristics into 5 categories: locally confined disease, biochemical recurrence, metastatic PCa - not otherwise specified (NOS), trials that include all prostate cancer patients regardless of disease characteristics, and castration-resistant PCa [9].

The goal of the trials was also evaluated according to the role of PET imaging and categorized in 6 classes: Post Treatment Follow-Up, Primary Tumor Evaluation, General Staging, General Staging with Emphasis on Nodal Status, Treatment Response Evaluation and Other or Unknown. In cases where trials fulfilled the criteria for more than one group, a trial was categorized in one of the categories by consensus (EVB and NC).

Primary sponsors were categorized as follows: Academic, Industry, State Sponsored, Collaborative Groups, and Foundations. Source of monetary support was determined by using the modified methodology of Hirsch et al. [10, 11] as described in Cihoric et al. [5].

To evaluate which disciplines, lead the registered studies on PET utilization in PCa we identified and analyzed the reported contact data of the “Overall Study Officials” (OSO). OSOs are defined as “Person(s) responsible for the overall scientific leadership of the protocol, including study principal investigator”. In a subsequent step we performed an online search of each identified OSO for their respective current affiliation and declared specialization. For the online search PubMed, Google Scholar and the generic Google search engine were utilized. If the OSO reported more than one specialization or board certification we selected the one that corresponded to their current institution.

Additionally, trials were analyzed across the reported design elements, current recruitment status and results availability.

## Results

At the time of data acquisition the majority of trials were in “active” recruitment status (*n* = 46, 34%) followed by trials that had been “completed” - (*n* = 34, 25%) and trials with “closed recruitment but active follow-up” (*n* = 23, 17%). Eleven (8%) trials were registered but did not start recruitment. Status was “unknown”, “available”, “enrolling by invitation” and “approved for marketing” in 8 (6%), 3 (2%), 3 (2%) and 1(1%) trial respectively. Seven (5%) trials were terminated, 3 trials due to insufficient recruitment, 1 due to lack of efficiency, 1 due to methodological reasons, 1 for “business reasons”, and 1 due to “the principal investigator leaving the institution”. One (1%) trial was suspended for preliminary data analysis.

The trials were conducted in 17 countries within 135 recruitment sites. United States (*n* = 82, 61%) and Canada (*n* = 17, 13%) were the countries with the most recruitment sites (*n* = 99, 73%), followed by Denmark (*n* = 6, 4%), the United Kingdom (*n* = 5, 4%), France (*n* = 4, 3%), Norway (n = 4, 3%) and Switzerland (*n* = 3, 2%). Austria, Finland, Italy and the Netherlands each had 2 (1%) recruitment sites. Australia, China, Germany, India, republic of Korea and Sweden had 1 (1%) recruitment site each. The characteristics of all 137 trials are summarized in Table 1.

**Table 1:** Clinical Trial Characteristics

Primary Purpose of a Trial		
Diagnostic	93	68%
NR^a^	20	15%
Treatment	17	12%
Screening	3	2%
Other	2	1%
Basic Science	2	1%
Trial has data monitoring comity?		
No	53	39%
Yes	60	44%
NR^a^	24	18%
Number of collaborators		
0	78	57%
1	43	31%
2	12	9%
3	4	3%
Number of recruitment centers per trial		
1	125	91%
2	2	1%
3	2	1%
NR^a^	8	6%
Trial duration in years - Median (Range)	3 years (1–21)	
Year of last database entry update		
≤ 2014	13	9%
2015	13	9%
2016	39	28%
2017	72	53%
Trials with results submitted to the ClinicalTrials.gov	14	10%
Number of patients		
0–25	47	34%
26–50	27	20%
50–100	22	16%
101–200	22	16%
200–300	10	7%
301–400	1	1%
> 401	4	3%
NR^a^	4	3%

^a^*NR* not registered

Interventional and expanded access studies comprised most of the clinical trials portfolio (*n* = 121, 88%). Most of the trials were in an early phase. Phase 1 and 2 comprised 46% of the complete trial portfolio. Forty-three (31%) trials did not contain information on the research phase. Interventional trials design data are presented in Table 2.

**Table 2:** Clinical Trial Design Data

Study type		
Interventional	117	97%
Expanded Access	4	3%
Trial phase		
NR	43	36%
Early Phase 1	6	5%
Phase 1	15	12%
Phase 1/Phase 2	13	11%
Phase 2	29	24%
Phase 2/Phase 3	7	6%
Phase 3	7	6%
Phase 4	1	1%
Allocation		
NR	113	93%
Non-Randomized	18	15%
Randomized	6	5%
Interventional model		
Single Group Assignment	102	84%
Parallel Assignment	14	12%
NR	20	17%
Crossover assignment	1	1%
Masking (Blinding)		
Open Label	96	79%
NR	21	17%
Masked (Blinded)	4	3%
Number of arms		
1	96	79%
2	11	9%
3	7	6%
NR	7	6%
Number of reported primary outcomes		
NR	4	
1	88	
2	11	
3	10	
≥ 4	8	
Number of reported secondary outcomes		
NR	44	
1	32	
2	13	
3	6	
≥ 4	26	

*NR* not regsitered

We have detected 16 (12%) observational trials, mostly prospective by nature of data collection (*n* = 14, 10%), one (1%) cross-sectional and one (1%) retrospective. Observational model was case-only in 7 (5%), cohort in 6 (4%) and case-control in 2 (1%). Thirteen (9%) trials have one observed group and 3 (2%) have two observed groups.

**Fig. 1:**
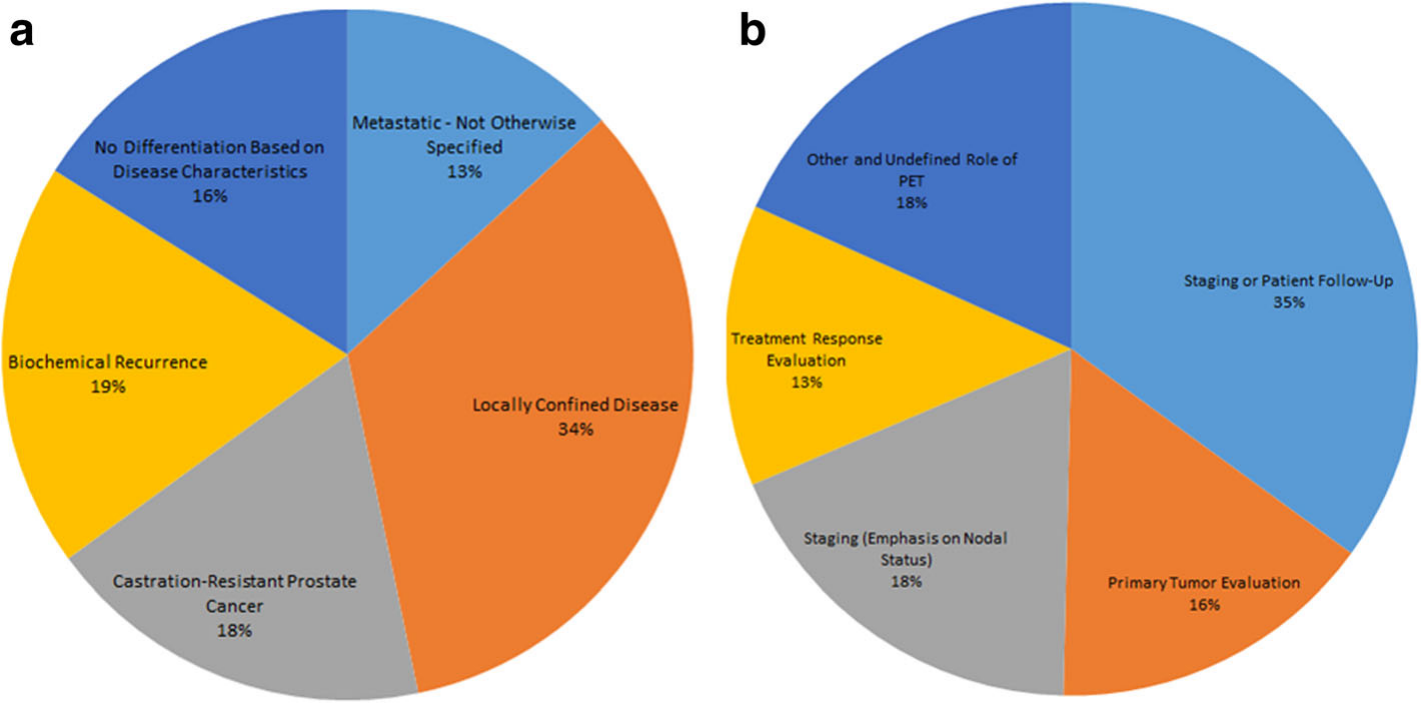
Distribution of trials based on disease characteristics and role of PET within a trial. (**a**) Trial distribution according to the disease characteristics. (**b**) Trial distribution according to the goal of PET examination

Locally confined disease was of major interest (*n* = 46, 34%), followed by metastatic disease – not otherwise specified (*n* = 43, 13%). Trial distribution according to the disease characteristics is shown in Fig. 1a. Primary tumor evaluation and staging with emphasis on nodal disease was the focus of 46 (34%) studies (Fig. 1b).

Evaluation of PET was the primary goal of the trial in 114 (83%) cases. In 22 trials (16%) PET evaluation or utilization was optional. Sixteen (12%) trials explicitly evaluated a combination of MRI and PET, and in 8 (6%) PET/MRI was optional. Other trials did not mention specific equipment (*n* = 113, 82%).

Most of the trials evaluated only one agent (*n* = 122, 89%), while 15 (11%) trials included more than one agent. Choline and PSMA represented the two major groups (total 50%) and were equally distributed across the trial portfolio with 25% (*n* = 34) each. Trials with other agents comprised 50% (*n* = 69) of the portfolio. Four (3%) trials did not report on the type of PET agent investigated. In total 5 different radionuclides were used for labeling, namely, ^11^C, ^18^F, ^68^Ga, ^89^Zr and ^64^Cu. Fifteen (11%) trials evaluated or allowed more than one PET agent within a study, but only 3 (2%) trials compared two agents directly. In total, 35 PET agents were evaluated. The list of all used agents is shown in Table 3.

**Table 3:** List of detected positron emission tomography agents

Positron Emission Tomography Agent as Stated in ClinicalTrials.gov	Radionuclid	IUPAC name of the carrier or other relevant source	Number of trials
Acetate	(11C)^a^	Acetate	22
Choline	(11C)^a^	2-Hydroxy-N,N,N-trimethylethan-1-aminium	16
Choline	(18F)^a^	2-Hydroxy-N,N,N-trimethylethan-1-aminium	37
Choline	NOS^a^	2-Hydroxy-N,N,N-trimethylethan-1-aminium	7
Citrate	(68Ga)	2-Hydroxypropane-1,2,3-tricarboxylic acid	1
DFO-huJ591	(89Zr)	Recombinant humanized monoclonal antibody J591 conjugated to chelator desferrioxamine B	1
DFO-MSTP2109A	(89Zr)	Desferrioxamine - DFO-MSTP2109A is an antibody that works against STEAP1 - found on the surface of prostate cancer cells	1
DHT	(18F)^a^	(5S,8R,9S,10S,13S,14S,17S)-17-hydroxy-10,13-dimethyl-1,2,4,5,6,7,8,9,11,12,14,15,16,17-tetradecahydrocyclopenta[a]phenanthren-3-one	3
DOTA-Bombesin	(68Ga)	68Ga-DOTA-Bombesin is a gallium-68-labeled gastrin-releasing peptide receptor (GRPr) antagonist. DOTA is [4,7,10-Tris-(carboxymethyl)-1,4,7,10-tetraazacyclododec-1-yl]-acetyl.	1
DOTATET	(68Ga)	1,4,7,10-tetraazacyclododecane-1,4,7,10-tetraacetic acid (also known as DOTA)	1
FAZA	(18F)	Nitroimidazole nucleoside analogue 1-(5-fluoro-5-deoxy-*α*-D-arabinofuranosyl)-2-nitroimidazole (FAZA)	2
FDG	(18F)^a^	2-Deoxy-2-[18F]fluoroglucose	15
FLT	(18F)	1-[(2R,4S,5R)-4-fluoro-5-(hydroxymethyl)oxolan-2-yl]-5-methylpyrimidine-2,4-dione	1
Fluciclovine	(18F)	Anti-1-amino-3-18F-fluorocyclobutane-1-carboxylic acid	13
Fluciclovine	(NOS)	Anti-1-amino-3-18F-fluorocyclobutane-1-carboxylic acid	1
Fluorocholine	(18F)	Fluoromethyl-(2-hydroxyethyl)-dimethylazanium;chloride	1
FMAU	(18F)	2′-deoxy-2′-[18F]fluoro-5-methyl-1-beta-D-arabinofuranosyluracil (fluorine F 18 d-FMAU [18F-FMAU]),	3
MeAIB	(NOS)^a^	2-methyl-2-(methylamino)propanoic acid	1
Methionine	(11C)^a^	(2S)-2-amino-4-methylsulfanylbutanoic acid	3
MISO	(18F)	1-methoxy-3-(2-nitroimidazol-1-yl)propan-2-ol	1
NaF	(18F)^a^	Sodium;fluoride	20
NODAGA-MJ9	(Ga-68)^a^	1,4,7-triazacyclononane,1-glutaric acid-4,7 acetic acid (NODAGA)	1
NOTA-BBN-RGD	(68Ga)	1,4,7-triazacyclononanetriacetic acid (NOTA), Arg-Gly-Asp (RGD) and bombesin (BBN)	1
P15–041	(68Ga)	Unknown	1
PACAP	(64Cu)	Pituitary adenylate cyclase-activating peptide	2
Peripheral Benzodiazepine Receptor-28	(11C)	Peripheral Benzodiazepine Receptor-28	1
PSMA DCFPyL	(18F)^a^	*N*-[*N*-[(*S*)-1,3-dicarboxypropyl]carbamoyl]-4-[^18^F]fluorobenzyl-L-cysteine ([^18^F]DCFBC)	21
PSMA	(68Ga)	Glutamate carboxypeptidase II, also known as N-acetyl-L-aspartyl-L-glutamate peptidase I	15
PSMA	(89Zr)	Glutamate carboxypeptidase II, also known as N-acetyl-L-aspartyl-L-glutamate peptidase I	2
PSMA CTT 1057	(NOS)	Cancer Targeted Technology (CTT), a privately held Seattle-based biotechnology firm that will develop the agent, CTT1057. CTT1057 is a small molecule that homes in and binds irreversibly to prostate specific membrane antigen (PSMA)	1
PSMA DCFBC	(18F)	(N-[N-[(S)-1,3-dicarboxypropyl]carbamoyl]-4-F-fluorobenzyl-L-cysteine) (F-DCFBC)	4
PSMA Df-IAB2M	(89Zr)	Zr-89-desferrioxamine-IAB2M	1
RM2	(68Ga)	68Ga-RM2 stands for Galium-68 labeled DOTA-4-amino-1-carboxymethylpiperidine-D-Phe-Gln-Trp-Ala-Val-Gly-His-Sta-Leu-NH2	3
Sodium Fluoride	(18F)	Sodium Fluoride	1
uPAR NOTA	(68Ga)	Urokinase Plasminogen Activator Receptor	1

^a^Agent was an optional or was compared to other agent in more than one trial

**Fig. 2:**
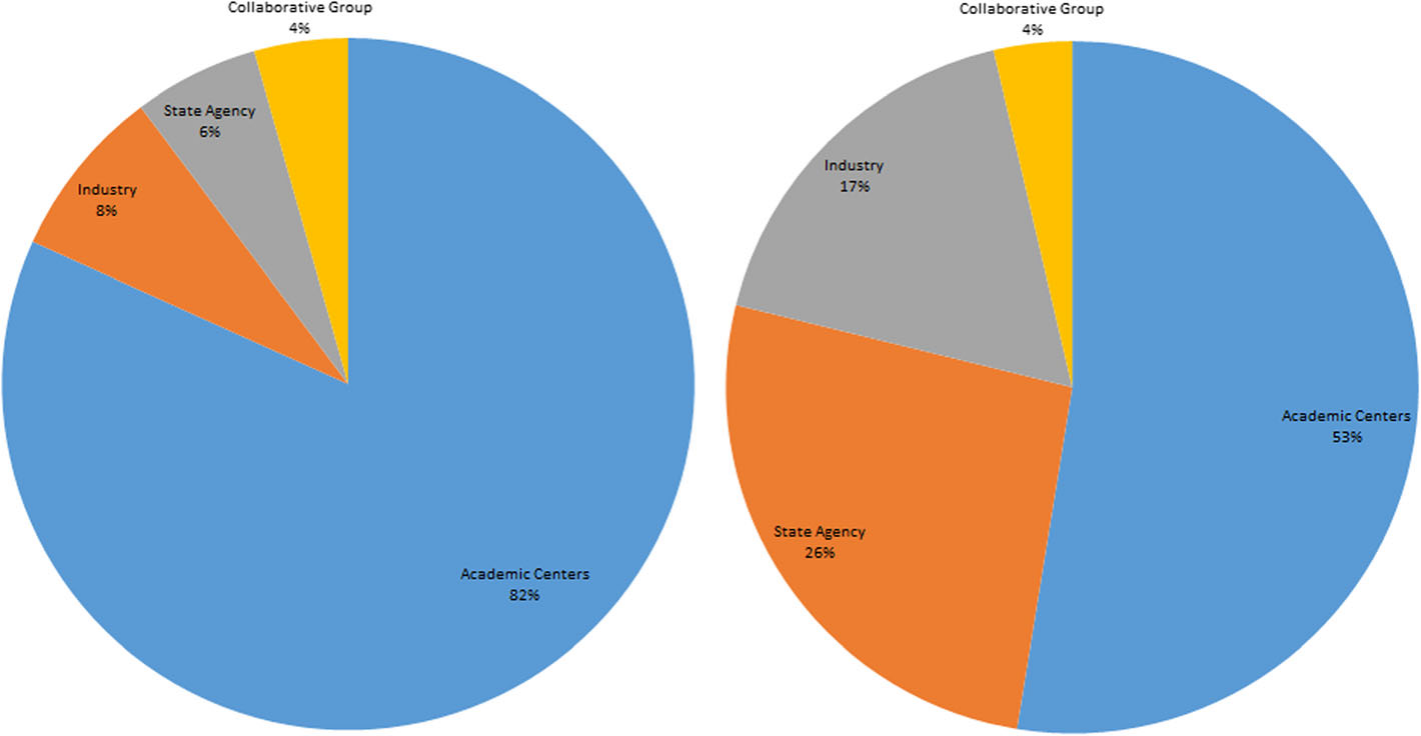
Primary sponsors and source of funding

**Fig. 3:**
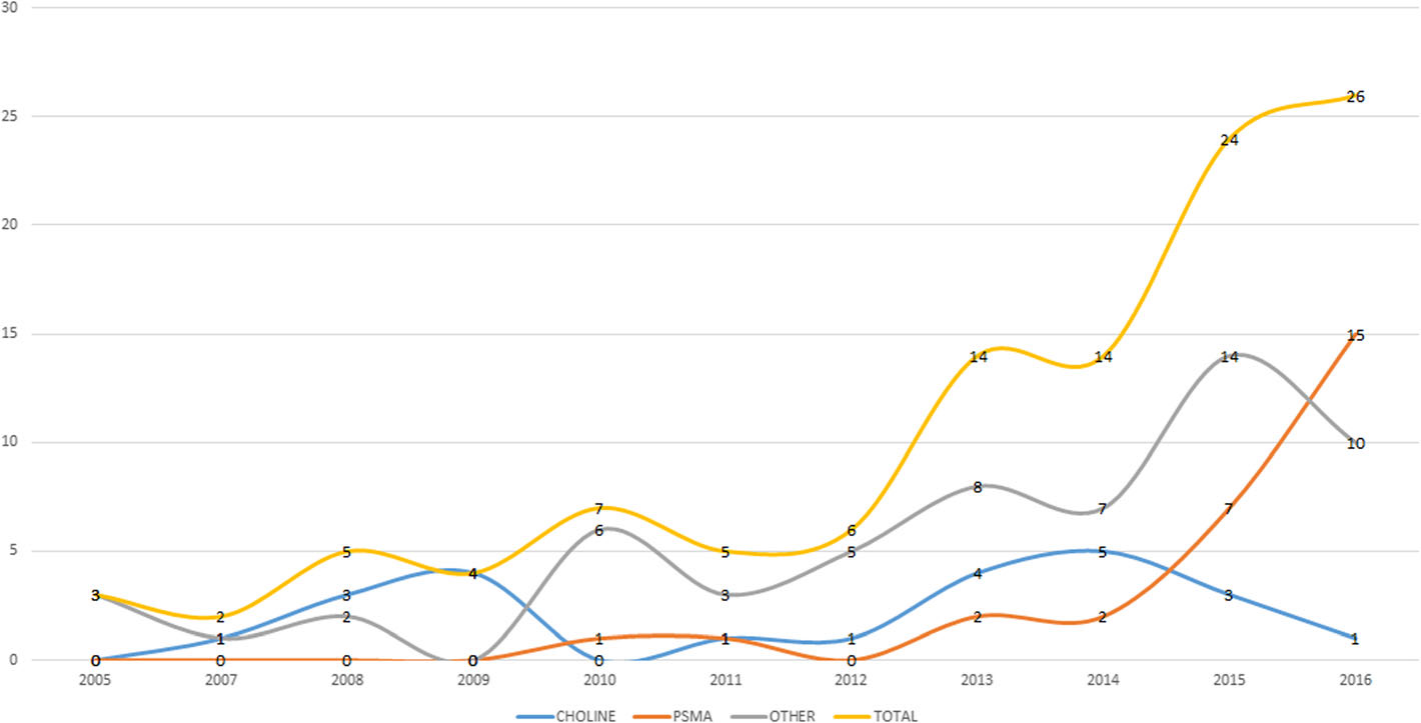
The number of newly registered trials per year according to an evaluated agent

PSMA trials showed the highest average annual growth rate of 56% followed by choline (44%) and other PET agents (7%). The average annual growth rate of newly started trials during 2005 and 2017 was 35%. The number of newly registered trials per year according to the evaluated agent is shown in Fig. 2. Academic centers were the most common primary sponsors (*n* = 112, 82%). The financial support from collaborative groups was limited to 5 trials only and state agencies acting as a co-funder of a study were detected in 28 trials (20%). Details on primary sponsors and source of funding are shown in Fig. 3.

74% (101) of all trials reported on the trial overall official (person responsible for overseeing of the protocol). Of those, the most common specialization was nuclear medicine (*n* = 38, 28%), followed by medical oncology (*n* = 25, 18%), radiology (*n* = 13, 9%), and radiation oncology (*n* = 12, 9%). Other specialties and non-clinical personnel were represented by 8 principal investigators. Urology was represented by 5 (4%) trial principal investigators.

## Discussion

Since its discovery, PET promised to be a powerful tool for cancer diagnostics and treatment. In a short amount of time, it managed to deliver and even exceeded the expectations in several diseases, with many notable examples in the field of oncology, such as lymphomas, lung or head and neck cancers [12–15].

There is an increased interest in integrating PET/CT in the management of PCa. Though initial attempts failed to bring the expected results thus integrating PET/CT in the diagnosis and treatment algorithms of PCa, the recent United States Food and Drug Administration (FDA) approval of fluciclovine (^18^F) as well as new advances such as the discovery of the Prostate-specific membrane antigen (PSMA), a well-characterized imaging biomarker, have brought this imaging modality back into the spotlight.

Our analysis showed that trials evaluating PET in PCa patients are predominantly early phase, single arm and open label, with more than 50% of all being small trials including less than 50 patients. It seems that the investigations in PET and PCa mimics those for the general oncological trials portfolio, where the early phase trials with low patient number dominate [10]. The issue of trial cost is well known, but this can be easily addressed by global collaborative efforts as it has with most tumor entities and imaging modalities. In the case of PET this is hard to achieve due to several reasons, such as the price of production and transport, regulatory environments and logistic reasons like the short life of positron emitters.

In addition to the general overview, specific trials deserve special mention. Two trials on fluciclovine PET/CT which have led to the FDA approval for diagnostic purposes in men with suspected PCa recurrence have a significant role in recognizing PET as a useful diagnostic method. The first trial compares fluciclovine PET/CT with the (111) In-capromab pendetide single photon emission computerized tomography. Results were controlled with biopsy (NCT00562315) [16]. The second one compared fluciclovine with choline PET. Fluciclovine was superior to choline in terms of staging of patients with biochemical relapse [17]. This makes fluciclovine the only tracer with high level evidence data and the de-facto standard for future trials in these clinical settings. Another noteworthy trial on fluciclovine is a retrospective observational trial with 714 patients sponsored by the manufacturer - Blue Earth Diagnostic (NCT02443571), where the primary endpoint is safety and secondary endpoint detection rate, sensitivity and specificity and negative predictive value in biochemically recurrent PC.

Phase III trials are of special interest, in general most evidence is generated from them and they serve as a polygon for eventual changes in clinical practice. In the case of PET and PCa, they are indeed rare. Of those registered in the ClinicalTrials.gov database, 3 trials evaluate choline, 6 PSMA based agents, 3 fluciclovine and 3 other agents. While the fluciclovine trials have already completed recruitment, PSMA phase III trials results are still recruiting. Completion of trials is expected in the coming years. One trial in 2018 - NCT02981368, one in 2023 - NCT03001869 and three in 2020 - NCT02659527, NCT02678351 and NCT02919111. Important characteristics of PSMA trials are more focused on local disease and nodal status. However, there is no direct comparison of the different agents or other PET tracers or diagnostic methods that may cover local and systemic disease.

However, the goal of our study is not to discuss the historical utilization of individual PET agents, but instead to shed some light and create an overview of current research efforts on a macro scale, which could potentially provide insights into possible future directions and research areas currently underresearched and in need of effort intensification.

Initiative for the trials on PET and PCa comes almost exclusively from academic institutions, hence the financial burden mostly lies on the shoulders of individual investigator centers. Having in mind financial burden and logistic limitation it is not highly probable that the academic community will conduct multi-centric phase III trial with a high number of patients for one specific therapy-oriented endpoint. Instead, the community may focus its efforts towards an organization of high quality observational trials. An observational trial, although precipitated as lower quality investigation, represents an important tool in addressing important medical questions [18]. The importance of observational trials and theoretical basis is well described in the work of Williams RJ et al. [19] and Choi BC et al. [20].

Our trial portfolio showed a lack of international collaborations. A potential solution, would be a conglomeration of the individual interest groups achieving both a reduction of expenses and a reduction of wasted resources. This includes detection of common interests, collaborative efforts and data sharing. A successful example of collaborative efforts in oncology, where results resulted to practice changing, is the EMBRACE Study – “An international study on MRI-guided Brachytherapy in locally Advanced Cervical cancer” [21].

An important aspect of a study is the detection of the growing interest in PET and prostate research. The growth in the number of newly registered trials may be explained by the strengthened regulatory environment when it comes to registration of interventional trials. However, ICMJE (International Committee of Medical Journal Editors) recommendations and regulations exist for over 10 years and interventional trial registration is obligatory in the USA since 2007. A significant quantitative “jump” is only seen during the last 3 years. This may be attributed to the ever-growing importance of metabolic imaging in diagnostics and treatment of PCa patients. It is also possible that public awareness is growing, and the scientific community is addressing the need for high quality data.

An interesting observation made during this study was a lack of any kind of comprehensive registry where the agents may be searched or evaluated across their type, mechanism of action or by any other means. The data entries in ClinicalTrials.gov are not sufficient and classification of the agents demands extensive manual work. The problem of resource identification and categorization is well recognized across other biomedical disciplines and it resulted in the Force11 initiative [22, 23].

Our analysis has some limitations including the possibility that some interventional prospective trials were not registered within the ClinicalTrials.gov or are not registered at all. However, ClinicalTrials.gov is the largest and most accurate registry to date, and we strongly believe that our results reflect the current state of current and past clinical trials worldwide. Furthermore, during trial classification we were forced to reduce the total number of possible combinations to a reasonable number of groups. For example, in one trial the investigators stated that the trial goal was staging in metastatic setting, but as an additional primary goal they correlated the tumor Gleason score with PET intensity. A complete annotated dataset is available for download (Additional file 1). Primary and secondary endpoints are also available (Additional file 2).

Despite these limitations, our analysis provides an accurate description of the scientific activity on the use of PET in clinical trials involving PCa patients.

## Conclusion

There is a growing interest in PET utilization in PCa. However, prospective trials investigating PET in PCa, especially those that may generate higher evidence level, are in general rare. Nevertheless, the scientific community is showing a strong and ever-growing interest in the field and we expect that in the coming years, more trials will be initiated ultimately delivering the required Level 1 evidence.

## Additional files

Additional file 1: Annotated Dataset. (XLSX 54 kb)

Additional file 2: Trials Endpoints. (XLSX 56 kb)

## Abbreviations

CT: Computed tomography
FDA: United States Food and Drug Administration
FDG: Fluorodesoxyglucose
MRI: Magnetic resonance imaging
NOS: Not otherwise specified
OSO: Overall Study Officials
PCa: Prostate cancer
PET: Positron emission tomography
PET/CT: Positron emission tomography combined with computed tomography
PET/MRI: Positron emission tomography combined with magnetic resonance imaging
PSMA: Prostate-specific membrane antigen
PSMA: Prostate-specific membrane antigen
WHO: World Health Organization
